# Low Dose Astaxanthin Treatments Trigger the Hormesis of Human Astroglioma Cells by Up-Regulating the Cyclin-Dependent Kinase and Down-Regulated the Tumor Suppressor Protein P53

**DOI:** 10.3390/biomedicines8100434

**Published:** 2020-10-19

**Authors:** Juhyun Shin, Ramesh Kumar Saini, Jae-Wook Oh

**Affiliations:** 1 Department of Stem Cell and Regenerative Biotechnology, Konkuk Institute of Technology, Konkuk University, 120 Neungdong-ro, Gwangjin-gu, Seoul 05029, Korea; junejhs@konkuk.ac.kr; 2 Institute of Natural Science and Agriculture, Konkuk University, Seoul 05029, Korea; saini1997@konkuk.ac.kr

**Keywords:** astaxanthin, glioblastoma multiforme, reactive oxygen species, superoxide dismutase, P53, apoptosis, hormesis

## Abstract

Astaxanthin (AXT) is a xanthophyll carotenoid known to have potent anti-cancer effects via upregulation of the intracellular reactive oxygen species (ROS) levels, which triggers apoptosis of cancer cells. While several studies have shown that AXT has potential as an anti-cancer drug, its effects in glioblastoma multiforme cells remain relatively unknown. In this study, we investigated the effects of AXT in the astroglioma cell lines U251-MG, T98G, and CRT-MG. We found that the response to AXT varied between cell lines. Moreover, U251-MG cells showed a specific hormetic response to AXT. At high concentrations (20–40 μM), AXT triggered apoptosis in U251-MG cells, as it has been previously shown in other cancer cell lines. However, low concentrations (4–8 μM) of AXT were found to upregulate the proliferative cell cycle. Furthermore, at low concentrations, AXT did not affect the intracellular ROS levels, while the superoxide dismutase activity increased moderately. Western blot analysis showed that treatment with a low concentration of AXT upregulated cyclin-dependent kinase (Cdk) 2 and p-Cdk2/3 levels and downregulated the expression of tumor protein p53. Thus, our results showed that AXT has a hormetic effect in the astroglioma cell line U251-MG.

## 1. Introduction

Astaxanthin (3, 3′-dihydroxy-β, β′-carotene-4, 4′-dione, AXT) is a xanthophyll carotenoid that was first identified in lobsters and can be found in various marine organisms [1], including the microalgae *Haematococcus pluvialis*, which is one of the major commercial sources of AXT [2]. However, it is also found in various surface-dwelling organisms, such as the yeast *Phaffia rhodozyma* and in propolis collected from bees [3]. AXT has recently become the focus of several studies as it has been shown to have multiple pharmacological benefits [4], namely anti-oxidant and anti-inflammatory effects [3,5,6]. Owing to these health benefits and its usefulness as a food colorant, the global market for AXT has been increasing rapidly and is expected to reach $2.57 billion by 2025 [7]. Interestingly, in vivo and in vitro studies of its effects on cancer suggest that administration of high doses of AXT leads to cell cycle arrest and that it has pro-apoptotic properties; however, these effects are highly dependent on the cell line analyzed (IC_50_ ranging from 39 to 720 µM depending on the cell line). This indicates that AXT could potentially be used as an anti-cancer agent [8]. The discrepancy between the response of healthy and cancer cells to AXT administration is probably due to the fact that over-proliferative cancer cells maintain high reactive oxygen species (ROS) levels when compared to healthy cells. In an environment with high ROS levels such as cancer cells, high levels of carotenoids act as pro-oxidants rather than anti-oxidants, leading to an imbalance in ROS expression, and thereby triggering apoptosis [9].

Glioblastoma multiforme (GBM) is the most common type of brain tumor, accounting for approximately 54% of brain cancers in the United States as of 2017 [10]. It is characterized by a poor prognosis, with the average survival time after diagnosis estimated to be approximately 15 months [11]. Currently, treatments for GBM are mostly based on surgical intervention, with temozolomide and radiation co-therapy leading to moderate improvements in patient outcomes [11]. Recently, the importance of micro RNAs as new approaches in the pathophysiology of brain tumors, including glioblastoma has been suggested [12]. One of the challenges in designing drug-based GBM treatments is the blood-brain barrier (BBB), which is important for maintaining homeostasis in the brain microenvironment but hinders the delivery of drugs [13]. It was previously shown that in rats, AXT can be detected in the hippocampus and cerebral cortex after oral administration, demonstrating that it has the ability to cross the BBB [14]. In vivo analysis has shown that AXT intake prevents pathological cellular stress in rat glioma cells [15]. Moreover, in healthy brain cells, AXT has been shown to have a neuroprotective effect against diseases such as cerebral ischemia, Parkinson’s, and Alzheimer’s disease [16], as well as to have potential as a geroneuroprotector [17]. However, studies have shown that AXT can trigger apoptosis by controlling redox homeostasis in various cancer cell lines, including oral, bladder, colon, liver, and lung cancer cell lines, as well as leukemia cell lines [8,18]. One study involving the GBM cell line A172 showed that AXT treatment did not trigger apoptosis up to a concentration of 150 µM but lowered the expression of matrix metallopeptidase proteins, and therefore, downregulated tumor cell invasion [19]. Although the effects of AXT on GBM remain relatively unknown, in addition to the evidence obtained from the other cancer cell lines mentioned above, the manipulation of redox homeostasis has already been shown to be an effective strategy for triggering apoptosis in GBM cells [20], suggesting that AXT has potential as a novel GBM treatment.

Hormesis is a toxicological term referring to a process in a cell that exhibits biphasic dose-response to a specific agent, characterized by a low dose beneficial effect and a high dose inhibitory effect [21]. Hormesis affects human health associated with nutritional [22] and medicinal [23] uptake. Particularly for anti-cancer drugs, in vitro experiments [24] and data analyses of patients [25] with lung and breast cancer [26] suggest that cancer cells of patients treated with anti-cancer drugs show a hormetic response to their respective drugs. This suggests that hormesis is a factor that should be considered while treating cancer patients in order to optimize treatment.

Here, we investigated the response of three GBM cell lines to AXT and found divergent responses in the three cell lines investigated. Notably, we reported, for the first time, that the U251-MG cell line showed a hormetic response to AXT treatment, with low AXT concentrations upregulating proliferation and high AXT concentrations triggering apoptosis.

## 2. Materials and Methods

### 2.1. Cell Lines and Culture Conditions

The astroglioma cell lines U251-MG and T98G were purchased from ATCC (American Type Culture Collection, Manassas, VA, USA). The CRT-MG cell line was established as previously described [27]. The U251-MG and T89G cell lines were grown in minimum essential medium (MEM) (Welgene, Daegu, Korea) supplemented with 1% of 100 mM sodium pyruvate (Welgene, Daegu, Korea), 1% of 100X penicillin/streptomycin (Welgene, Daegu, Korea), and 10% fetal bovine serum (FBS) (Welgene, Daegu, Korea). CRT-MG cells were grown in Dulbecco’s Modified Eagle Medium (DMEM) (Welgene, Daegu, Korea) supplemented with 1% of 100X penicillin/streptomycin (Welgene, Daegu, Korea) and 10% FBS. Cells were cultured in 100 mm cell culture dishes in an incubator at 37 °C and 5% CO_2_ and sub-cultured every 3 days after they reached a confluence of ~90%. The cells were not cultured beyond 15 passages.

### 2.2. Cell Viability Assay

The astroglioma cell lines U251-MG, CRT-MG, and T98G were plated in 96-well plates at a density of 5 × 10^3^ cells/well. After overnight incubation, the medium was changed to serum-free medium and the cells were incubated with corresponding concentrations of AXT (from *Blakeslea trispora*; Merck KGaA, Darmstadt, Germany) diluted in ethanol (EtOH): tetrahydrofuran (THF) = 3:1 *v/v*, for 24, 48, or 72 h. After treatment, WST-1 assay for cell survival was performed by adding 10 µL of EZ-Cytox (DoGen, Seoul, Korea) to 90 µL of serum-free medium and incubating the cells for an additional 1 h at 37 °C. Absorbance at 450 nm and reference absorbance at 600 nm were measured using a microplate reader (Bio-Tek, Winooski, VT, USA) and read using the Gen5 software (Bio-Tek).

### 2.3. ROS and Superoxide Dismutase (SOD) Assay

U251-MG cells were seeded in 6-well plates at a density of 2 × 10^5^ cells/well and treated with AXT at a concentration of 4–8 (low) and 20–40 (high) µM for 24 h in serum-free media. For the SOD assay, cells were harvested, resuspended in 200 µL of Dulbecco’s phosphate-buffered saline (DPBS) (Welgene, Daegu, Korea), and lysed by sonication. After isolation of the cell lysate supernatant by centrifugation, SOD activity was assayed using a SOD assay kit (Biomax, Seoul, Korea) and the total protein concentration in the supernatants was measured using a DC protein assay (Bio-Rad, Hercules, CA, USA). SOD activity was normalized to the total protein concentration for each treatment condition. For the ROS assay, cells were harvested, washed three times in DBPS, and resuspended in 500 µL of DPBS supplemented with 20 µM of 2ʹ, 7ʹ-dichlorofluorescin diacetate (DCFH-DA) (Enzo, Farmingdale, NY, USA) diluted in dimethyl sulfoxide (DMSO) (Merck-Millipore, Burlington, MA, USA) at a stock concentration of 200 mM. Cells were then incubated at 37 °C for 1 h before flow cytometry analysis (ACEA, San Diego, CA, USA) for FITC intensity.

### 2.4. Cell Cycle Analysis

U251-MG cells were seeded in 6-well plates at a density of 2 × 10^5^ cells/well and treated with AXT at a concentration of 0, 4, 8, 10, or 20 µM for 24 h in serum-free media. Cells were then harvested and fixed in 70% EtOH at 4 °C for 30 min. Fixed cells were resuspended in DPBS and treated with 50 µg/mL of propidium iodide (PI) (Molecular Probes, Eugenes, OR, USA). The cells were then kept in the dark on ice until the PI intensity was measured by flow cytometry.

### 2.5. Immunoblotting Assay

U251-MG cells were seeded in 6-well plates at a density of 2 × 10^5^ cells/well and treated with AXT at a concentration of 0, 4, 8, 10 or 20 µM for 24 h in serum-free media. Cells were washed once with DPBS before lysis in RIPA buffer (Merck-Millipore). The total protein concentration was determined using the DC protein assay (Bio-Rad) and was quantified by measuring absorbance at 750 nm using a microplate reader (Bio-Tek), according to the manufacturer’s instructions. Ten µg of total protein from each sample was fractioned by sodium dodecyl sulfate-polyacrylamide gel electrophoresis and transferred onto PVDF membranes. Following blocking for 1 h in 5% non-fat milk at room temperature (RT), the membranes were incubated with specific antibodies for 16 h at 4°C. Anti-p53 antibody (#OP03-100UGCN) was purchased from Merck-Millipore, anti-cyclin dependent kinase (Cdk) 2 (#sc-163), anti-phospho-Cdk2/3 (p-Cdk2/3) (#sc-12914), and anti-actin (#sc-1616) antibodies were purchased from Santa Cruz (TX, USA). After incubation with the primary antibodies at recommended dilution in 5% non-fat milk, the membranes incubated with HRP-conjugated secondary antibodies at RT for 2 h. The bands were detected using an enhanced chemiluminescence kit (Bio-Rad). The band intensities were measured using the ImageJ software [28].

### 2.6. Statistical Analysis

Statistical significance was evaluated using the two-tailed Student’s *t*-test. *P* values for the experiments were calculated from at least two independent experiments. The fluorescence-activated cell sorter (FACS) analyses were performed for 10,000 cells and experiments were repeated for three biological replicates.

## 3. Results

### 3.1. AXT Exhibits Hormesis in the U251-MG Cell Line

**Figure 1:**
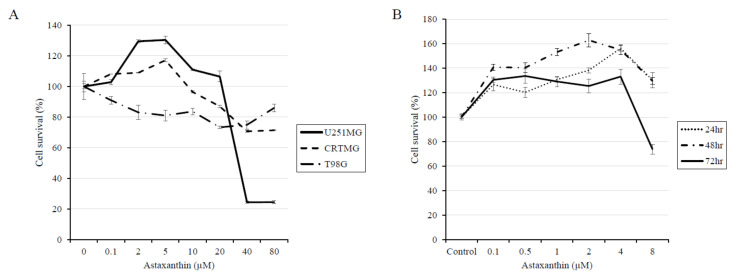
GBM cell lines show a hormetic effect in a dose and time-dependent manner. Cell survival in the GBM cell lines U251-MG, CRT-MG, and T98G was measured using the WST-1 assay after 24 h of treatment with corresponding AXT concentrations in serum-free media (**A**). Time-dependent proliferation and survival of U251-MG cells after treatment with corresponding AXT concentrations for 24, 48, and 72 h. (**B**) Error bars show the standard deviation obtained from more than three replicates (*n* > 3).

The effect of AXT in the three astroglioma cell lines U251-MG, CRT-MG, and T98G were assayed via the 4-[3-(4-Iodophenyl)-2-(4-nitro-phenyl)-2H-5-tetrazolio]-1,3-benzene sulfonate-1 (WST-1) assay (Figure 1A). At concentrations up to 80 µM, only the U251-MG cell line showed a significant response to AXT treatment, with an IC_50_ value of 25.4 µM after 24 h of treatment for U251-MG cells plated at a density of 5 × 10^3^ cells/well in 96-well plates, according to The Quest Graph™ IC_50_ Calculator (https://www.aatbio.com/tools/ic50-calculator). CRT-MG and T98G cell survival at the maximum AXT concentration used in this experiment was 71.3 ± 0.3% (*p* = 0.002 compared to the control) and 86.0 ± 2.4% (*p* = 0.152 compared to the control), respectively, suggesting that AXT might be effective at higher doses for CRT-MG cells and have only minor effects on T98G cells. These results suggest that AXT affects different astroglioma cell lines differently. Surprisingly, we found that U251-MG cells show a biphasic response to AXT, that is low doses of AXT have a proliferative effect, with a maximum survival increase of 130.4 ± 2.4% after treatment with 5 µM of AXT, while AXT concentrations over 20 µM have an apoptotic effect. To confirm these findings, we evaluated the effects of treatment with a low dose of AXT (0–8 µM) at various time points (Figure 1B). We once again observed a hormetic effect, with a proliferative response after 24 h of treatment, while longer treatments resulted in apoptosis. These results show that AXT treatment has a hormetic effect in U251-MG cells in a dose- and time-dependent manner.

### 3.2. AXT Induces Cell Proliferation at Low Concentrations

**Figure 2:**
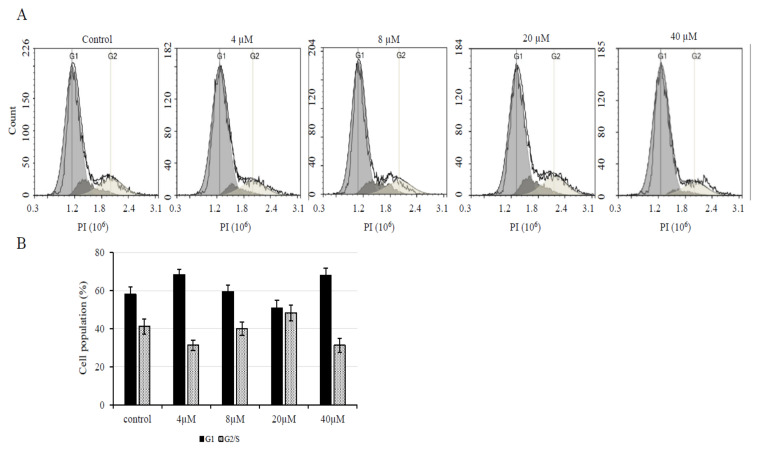
Treatment with low AXT concentrations induces cell proliferation. After AXT treatment, cells were fixed in ethanol and PI at a final concentration of 50 µg/mL was used to stain the nucleus. FACS was used to analyze the cell cycle in 10,000 cells for each treatment condition (**A**). Results were represented as % of cells and were plotted on a graph (**B**). Standard deviation was calculated from three replicates (*n* = 3).

In order to confirm that treatment with low concentrations of AXT induces cell proliferation in U251-MG cells, we analyzed the cell cycle via FACS. U251-MG cells were incubated for 24 h with 0, 4, 8, and 20 µM of AXT (Figure 2A). As mentioned previously, the AXT IC_50_ value for U251-MG cells was calculated to be 25.4 µM in treatments performed in 96-well plates. However, we did not observe any cytotoxic effects for treatments with AXT concentrations up to 20 µM in this specific experiment. This is probably due to the fact that the cells were plated at a higher density (2 × 10^5^ cells/well) in 6-well plates and it was previously reported that cell density might affect IC_50_ values [29]. We found no statistically significant decrease or increase in the number of cells in the G1 phase or S/G2 phase (*p* > 0.01), respectively, upon treatment with low AXT concentrations. However, the population of G1 cells decreased while the population of S/G2 cells increased in a dose-dependent manner after treatment with AXT at concentrations up to 10 µM, while the ratio between the two populations was returned to values similar to the control upon treatment at high concentration (40 µM) (Figure 2B). This suggests that low concentrations of AXT trigger cell proliferation and that this proliferative effect is lost after a threshold concentration is reached.

### 3.3. Antioxidant Activity of Enzymes Is Upregulated in U251-MG Treated With Low Concentrations of AXT

**Figure 3:**
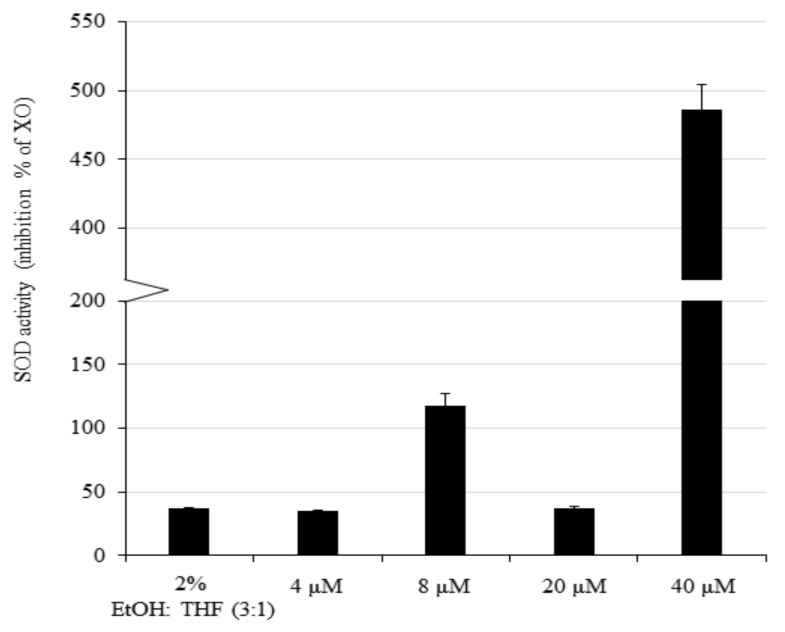
SOD activity in U251-MG cells is upregulated at low and high AXT concentrations. U251-MG cells were treated with AXT at a concentration of 4–8 (low) and 20–40 (high) µM for 24 h in serum-free media. SOD activity was measured using a SOD assay kit and was normalized to the total protein concentration for each treatment condition. Error bars shows the standard deviation obtained from two different replicates (*n* = 2). Experiments were performed in three biological replicates and the representative result of one experiment is presented.

It has been previously reported that SODs are upregulated after mild stress in diverse animal organisms, which triggers an adaptive stress response and hormesis [30]. In addition, AXT can trigger the activity of antioxidant SOD enzymes in normal cells [6]. On the other hand, AXT effects on cancer cells are contradictory. For example, in the colorectal cancer cell line LS-180, SOD activity was found to be upregulated [31], while in the breast cancer cell line SKBR3, *SOD1* was found to be downregulated and *SOD2* upregulated [32]. Therefore, we investigated SOD activity after AXT treatment in U251-MG cells (Figure 3). In U251-MG cells, treatment with the highest concentration of AXT was found to increase SOD activity by more than four-fold, and treatment with one of the low concentrations (8 µM) moderately increased SOD activity. While we only investigated four concentrations, results were consistent in three separate experiments performed. Therefore, our data suggest that in the GBM cell line U251-MG, AXT treatment triggers SOD activity after treatment of AXT in at least one low and one high concentration assayed.

### 3.4. Treatment with High AXT Concentrations Increased Intracellular ROS Levels while Low AXT Concentrations did not Affect ROS Levels

**Figure 4:**
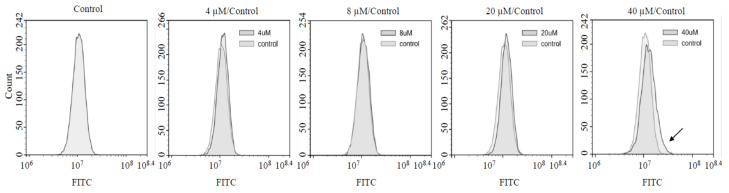
Low AXT concentrations upregulate SOD activity but not peroxidase levels, while high AXT concentrations trigger both SOD and ROS upregulation. U251-MG cells were treated with AXT at concentrations of 4–8 (low) and 20–40 (high) µM for 24 h in serum-free media. The cells were then incubated with 20 µM of DCFH-DA. The results of the control were used to compare FITC intensity changes determined by FACS. The same number of cells (10,000) was used in the analysis of all treatment conditions. Experiments were performed in more than three replicates (*n* > 3) and a representative graph is presented.

We further investigated if ROS levels are affected by AXT treatment, as the anti-cancer effects of AXT were correlated with an increase of intracellular ROS levels in previous studies [9,18]. We treated cells with AXT under the same conditions that we used to investigate SOD expression and performed the DCFH-DA assay to monitor intracellular ROS levels (Figure 4). Interestingly, we found no change in intracellular ROS levels, even at the concentration (8 µM) that triggered SOD upregulation. ROS levels increased after treatment with 40 µM of AXT. This suggests that antioxidant activity increases after treatment with a low concentration of AXT and that a higher threshold concentration of AXT has to be reached before it induces increase of intracellular ROS levels in U251-MG cells.

### 3.5. AXT treatment in U251-MG Cells Decreases p53 Levels, while a Low Dose of AXT Triggers Cdk2 and p-Cdk2/3 Upregulation

**Figure 5:**
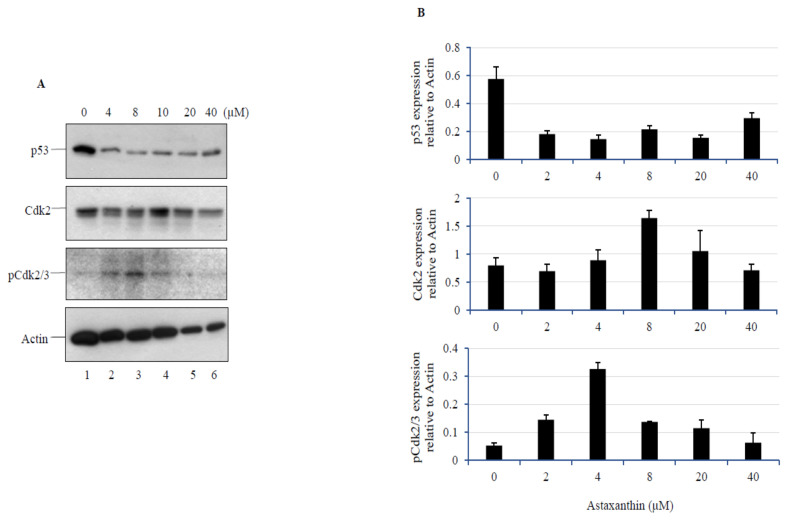
Western blot analysis after AXT treatment. After treatment with AXT at concentrations of 4, 8, 10, 20, and 40 µM for 24 h with control (1% EtOH:THF, 3:1), immunoblot analysis was performed using specific antibodies against p53, Cdk2, p-Cdk2/3, and actin. (**A**). Band intensity, normalized to actin, is shown (**B**). The western blot analysis was performed in three different replicates and the standard deviation was calculated based on those results (*n* = 3).

As treatment with low concentrations of AXT was found to affect cell cycle, we assessed p53, Cdk2, and p-Cdk2/3 expression levels after AXT treatment (Figure 5). Interestingly, p53 was found to be downregulated at all concentrations investigated, suggesting that in U251-MG cells, p53 expression can be controlled by AXT at low concentrations. While p53 expression was significantly downregulated, Cdk2 and p-Cdk2/3 levels were found to be slightly increased within the low concentration range, in a pattern similar to the results of the SOD activity assay in this study.

## 4. Discussion

AXT is a xanthophyll carotenoid, or termed as secondary carotenoid, as it is largely produced only under stress conditions (i.e., high irradiance and nitrogen limitation) by *chlorella zofingiensis*, *H. pluvialis*, and certain other species of green algae [4]. AXT has been shown to have high antioxidant activity compared to other carotenoids [33]. In cancer cell lines, it showed an opposite effect, acting as a pro-oxidant that induced apoptosis [8,18], which is a known phenomenon for carotenoids, probably due to the different ROS status between healthy and cancer cells [9]. In this study, we assayed the response of astroglioma cell lines to AXT treatment at concentrations ranging from 0 to 40 µM. Within this range, we observed that the cell response to AXT varied between cell lines, and we reported, for the first time, that AXT has a hormetic effect on U251-MG. While cell cycle changes were not statistically significant, survival data and western result suggest that proliferation pathway is triggered at molecular level at low concentration. From the three cell lines investigated, U251-MG and T98G exhibited p53 mutations [34] but had a divergent mutation status in the key locus [35]. CRT-MG is a cell line derived from a surgical specimen [27] and its mutation profile is yet to be analyzed. Previous reports have shown that U251-GM and CRT-MG cells show a similar response to CaCl_2_ and H_2_O_2_ treatment [36]; however, other similarities and differences between the two cell lines are yet to be analyzed. From the three astroglioma cell lines studied, our results showed that AXT had a hormetic effect only on U251-MG cells, demonstrating the limitations of in vitro studies for evaluating the effect of anti-cancer drugs in patients [37]. Nonetheless, this is, to our knowledge, the first report that showed that AXT might have a hormetic effect on cancer cells.

The proliferation of cells is regulated by p53, which controls Cdk phosphorylation (activation), thereby signaling the transition from the G1 phase to the S phase in the cell cycle [38]. As expected, we observed that treatment with low concentrations of AXT increased the population of cells in the G2/S phase in a dose-dependent manner. Moreover, treatment with low concentrations of AXT downregulated p53, while it upregulated Cdk2 and p-Cdk2/3. While U251-MG cells harbor a mutated form of p53 [39] that shows higher resistance to chemotherapy and radiotherapy compared to wild-type p53 [40], p53 downregulation in cancer cells is usually linked to proliferation [41]. Till date, carotenoids have been reported to upregulate p53 in cancer cell lines [42,43,44]. For example, the carotenoid lutein has been shown to inhibit breast cancer cell growth and increase p53 expression [45]. However, in healthy cells, carotenoids usually exhibit a protective effect against oxidative stress, and therefore, induce p53 downregulation, as seen in the corneal epithelial cells [46]. In a previous review, we suggested that this discrepancy might be dependent on the cellular environment; particularly, on its redox status [9]. Here, we found an instance of a biphasic response that was dose-dependent, suggesting that diverse factors could be involved in defining the role of carotenoids in cells.

Interestingly, we found that SOD activity showed a small increase (peak) at low concentrations of AXT, which was insufficient to trigger an increase in the intracellular ROS levels. SODs are enzymes that play an important role in antioxidant defense [47]. Therefore, this hormetic effect showed that treatment with low concentrations of anti-cancer drugs that raise ROS levels might have a negative effect. Indeed, a study in esophageal squamous cell carcinoma suggested that an increase in the SOD-2 levels resulted in resistance to cisplatin in the related cells [48]. ROS control is considered to be an effective cancer treatment method [49]. However, our data suggest that cellular uptake should be carefully considered, as it might have outcomes opposite to those intended.

In this study, we found that p53 was downregulated by treatment with AXT at low concentrations of up to 40 µM. Based on the results obtained for the biphasic hormetic response, we focused on the low concentration-induced proliferative response, as this is not usually observed in cancer cell lines. Further analysis should be done at higher AXT concentrations to evaluate the expression of p53 and other apoptotic proteins, such as caspases, in order to elucidate the apoptotic mechanism induced by AXT in GBM cell lines at higher concentrations.

Despite medical advancements, treating brain tumor including glioblastoma is still challenging. A number of factors make brain tumor a challenging disease to treat, including the brain’s natural defenses such as BBB, accessibility of the tumors and their ability to spread rapidly and the complexity of brain tumor. The ability of these tumors to resist almost all conventional and novel treatments relates to the unique microenvironmental properties of brain tissues. Recently, there have been reports that micro RNA plays an important role in controlling the chemosensitivity of brain tumors [12,50]. We applied marine natural substances to brain tumors in a new attempt in this study and report the results here. As such, new attempts are under way in this field.
